# Identification and Validation of Evolutionarily Conserved Unusually Short Pre-mRNA Introns in the Human Genome

**DOI:** 10.3390/ijms160510376

**Published:** 2015-05-07

**Authors:** Makoto K. Shimada, Noriko Sasaki-Haraguchi, Akila Mayeda

**Affiliations:** 1 Division of Gene Expression Mechanism, Institute for Comprehensive Medical Science, Fujita Health University, Toyoake, Aichi 470-1192, Japan; 2 Matsunami Research Park (Sosai-kouseikai), 10 Izumi-cho, Kasamatsu-cho, Hashima-gun, Gifu 501-6061, Japan

**Keywords:** G-rich sequence, H-InvDB, human genome, human transcriptome, intron length, pre-mRNA splicing, splice sites, ultra-short intron

## Abstract

According to the length distribution of human introns, there is a large population of short introns with a threshold of 65 nucleotides (nt) and a peak at 85 nt. Using human genome and transcriptome databases, we investigated the introns shorter than 66 nt, termed ultra-short introns, the identities of which are scarcely known. Here, we provide for the first time a list of *bona fide* human ultra-short introns, which have never been characterized elsewhere. By conducting BLAST searches of the databases, we screened 22 introns (37–65 nt) with conserved lengths and sequences among closely related species. We then provide experimental and bioinformatic evidence for the splicing of 15 introns, of which 12 introns were remarkably G-rich and 9 introns contained completely inefficient splice sites and/or branch sites. These unorthodox characteristics of ultra-short introns suggest that there are unknown splicing mechanisms that differ from the well-established mechanism.

## 1. Introduction

Pre-mRNA splicing is an essential process in eukaryotic gene expression. In higher vertebrates, however, the length of the target introns that need to be recognized vary markedly, from <50 nucleotides (nt) to >500,000 nt with degenerated splicing signals, or 5′ and 3′ splice sites at the ends of the introns. Interestingly, there are two statistical modes in the length distribution of eukaryotic introns; in humans, there is a narrow distribution of short introns with a peak at ~90 nt and a broad distribution of long introns with a peak at ~2000 nt [1,2,3].

The splicing mechanism was established using model pre-mRNA containing single short introns (one to several hundred nt), which are very efficiently spliced *in vivo* and *in vitro*. According to this system, the essential splicing sequences in pre-mRNA, namely the 5′ splice site, the branch-site sequence, and the 3′ splice site, are simultaneously bound by the splicing factors U1 snRNP, U2 snRNP, and U2AF^65^/U2AF^35^, respectively, leading to early ATP-dependent formation of the spliceosomal A complex (reviewed in [4,5]). Electron microscopy analysis has resolved the structure of the A complex, revealing an asymmetric globular shape with dimensions of ~26 nm × 20 nm × 19.5 nm [6]. The A complex fully occupies the length of a 79–125-nt single-stranded RNA sequence (equivalent to 4050–4800-nt RNA per μm; [7]). Nevertheless, there are many introns in the human transcriptome that are much shorter than 79 nt, and we have shown that specific human pre-mRNAs with 43-nt, 49-nt, and 56-nt introns are spliced *in vivo* and *in vitro* [8]. Therefore, we propose the following question: How are these tiny introns recognized by the known essential factors without steric hindrance?

To address this question, it is essential to identify and verify *bona fide* tiny human introns that are actually spliced. There are currently no objective- or evidence-based rules to determine the minimum intron length in the gene annotation procedure. We could list 22 evolutionarily conserved introns that are shorter than the 66-nt threshold, which we termed ultra-short introns. These ultra-short introns have not been characterized elsewhere. We then confirmed 15 introns with mRNA-Seq data, database annotations, and experimental evidence obtained by reverse transcription–polymerase chain reaction (RT–PCR) analysis. We demonstrated that nine ultra-short introns are actually spliced out, a process that is often required to escape from nonsense-mediated mRNA decay (NMD), suggesting that splicing of these ultra-short introns is a prerequisite for the expression of the host genes.

## 2. Results and Discussion

### 2.1. Length Distribution of Human Introns

**Figure 1:**
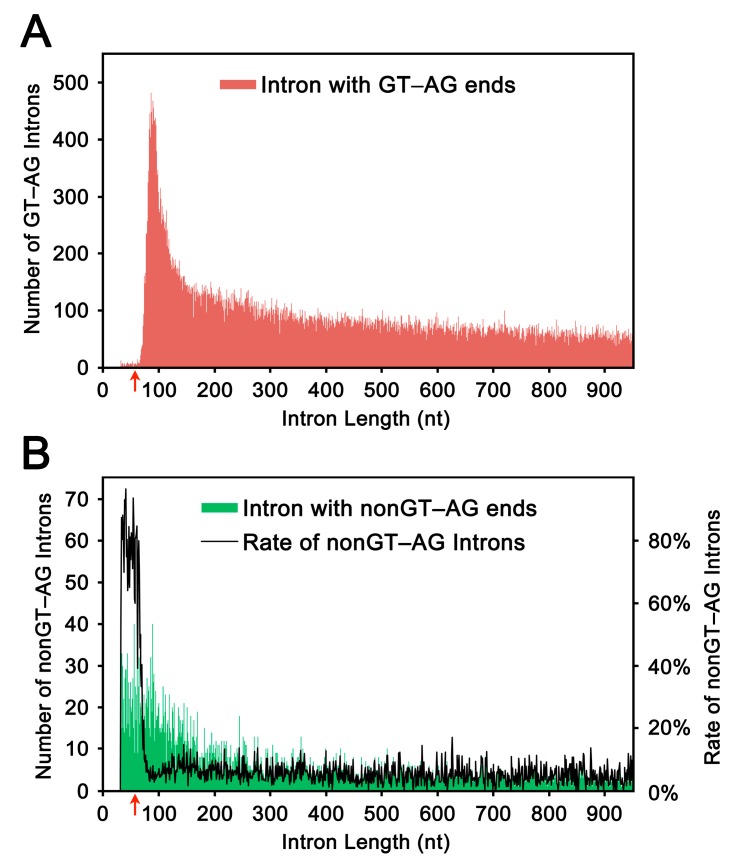
The length distribution of human introns (≤949 nt). (**A**) The lengths of introns with terminal GT and AG bases were plotted (red shading) based on the calculated lengths using H-InvDB annotation. The red arrow indicates the threshold (65 nt) from which the number of introns with GT–AG increases drastically toward the first peak at 83 nt; and (**B**) The lengths of introns with terminal non-GT and non-AG bases were plotted (green shading) in the same way. The rate of the number of non-GT–AG introns to the number of GT–AG introns was plotted with black line. The red arrow indicates the threshold (65 nt) from which the ratios of non-GT–AG introns to GT–AG introns decreased markedly.

Using sequence data deposited in a human annotated transcriptome database (H-InvDB), we plotted the distribution of intron lengths (Figure 1). All of the introns were selected from introns with authentic GT–AG ends. We observed a marked increase in the number of short introns from ~65 nt to a peak at ~85 nt (Figure 1A); this pattern is essentially consistent with the previously reported pattern [1,2,3,9]. We also plotted the distribution of introns with non-GT–AG ends (Figure 1B, green shading). Because the minor group introns, or U12-type introns, often possess non-GT–AG ends (reviewed in [10]), these atypical introns might contain U12-type introns. However, the apparent increase in the ratio of non-GT–AG introns to GT–AG introns for introns of ≤65 nt (Figure 1B, black line) evidently reflects false introns, or artifactual gaps, generated by the automatic alignment procedures. Based on these observations, we set the threshold at 65 nt, and defined the introns of ≤65 nt as ultra-short introns and those of 66–85 nt as short introns.

The authenticity of introns in the ultra-short range has never been investigated. Here, we validated the candidate introns in the ultra-short range to make a list of genuine ultra-short introns.

### 2.2. Selection and Validation of the Ultra-Short Introns

To select unequivocal introns in the ultra-short range, we performed three screening steps in 4528 tentative intronic sequences retrieved from H-InvDB. Using a human mRNA/cDNA sequence database, we first eliminated the transcripts that were erroneously mapped in H-InvDB with insertions/deletions to the genomic sequence. From the resulting 4027 candidate introns, conserved introns were selected by two kinds of BLAST searches: one targeted a genomic database using flanking exons of the candidate introns as queries, and the other targeted a transcriptome database using concatenated flanking exons as queries. Only candidate introns for which the results of both BLAST searches contained the same species with high similarity scores were selected. As a result, we obtained 1253 short introns (37–85 nt), of which 23 were in the ultra-short range (37–65 nt). By removing one ultra-short intron, which was a partial transcript, 22 candidate introns were finally determined as conserved ultra-short introns (Table 1).

**Table 1:** Candidate of human introns (≤65 nt) conserved in both genome and transcriptome sequences.

SN a	Length (nt) ^b^	ID number of HIT ^c^	Intron number d	Total no. of introns ^e^	Site of intron	Data in Ensembl ^f^	AA-seq g	Intron frequency ^h^	RT–PCR analysis * i	RNA-Seq data * j	Individually sequenced * k	Confirm. l	ID number of HIX ^m^	Host gene (HGNC) ^n^
**1**	37	HIT000059291	1	3	CDS	Yes	I	1/2	Expressed	No	**Yes**	**Yes**	HIX0029777	AQP12A
2	41	HIT000276161	4	4	CDS	Yes	II	1/23	Expressed	No	RM	No	HIX0001032	ENSA
**3**	43	HIT000008845	6	14	CDS	Yes	I	1/4	**Spliced**	**Yes**	No	**Yes**	HIX0013170	ESRP2
**4**	47	HIT000325704	2	15	CDS	Yes	II	1/1	No-Exp	No	**Yes**	**Yes**	HIX0003317	IFRD2
**5**	49	HIT000009363	12	13	CDS	Yes	I	3/11	**Spliced**	**Yes**	No	**Yes**	HIX0023123	NDOR1
6	50	HIT000084762	8	10	CDS	Yes	III	1/13	Expressed	No	No	No	HIX0022245	SAMD14
**7**	54	HIT000325704	3	15	CDS	Yes	II	1/1	Expressed	No	**Yes**	**Yes**	HIX0003317	IFRD2
8	54	HIT000333308	1	2	CDS	No	VII	1/1	Expressed	No	No	No	HIX0059400	HSP90B2P
9	55	HIT000278575	1	5	CDS	No	IV	1/7	Expressed	No	RM	No	HIX0006057	AKIRIN2
**10**	56	HIT000192494	7	13	CDS	Yes	I	9/10	**Spliced**	**Yes**	**Yes**	**Yes**	HIX0005482	HNRNPH1
11	61	HIT000302202	1	13	5′ UTR	Yes	I	1/15	Expressed	No	RM	No	HIX0001133	MSTO1
**12**	62	HIT000279220	1	7	CDS	Yes	I	9/11	**Spliced**	**Yes**	**Yes**	**Yes**	HIX0027515	SIGLEC6
13	62	HIT000333305	1	2	CDS	Yes	II	1/1	Expressed	No	No	No	HIX0202199	HSP90AB4P
**14**	62	HIT000495960	1	6	CDS	Yes	II	5/5	**Spliced**	**Yes**	No	**Yes**	HIX0202884	SIGLECP3
**15**	63	HIT000191419	3	4	CDS	Yes	I	3/3	n/a	**Yes**	No	**Yes**	HIX0079411	PRH1
16	63	HIT000091849	1	2	5′ UTR	Yes	VI	1/1	Expressed	No	No	No	HIX0036362	–
**17**	65	HIT000324311	10	28	CDS	Yes	II	1/1	**Spliced**	**Yes**	**Yes**	**Yes**	HIX0003640	PLXNA1
**18**	65	HIT000058074	1	20	CDS	Yes	I	1/4	No-PCR	**Yes**	**Yes**	**Yes**	HIX0034231	RECQL4
**19**	65	HIT000052133	11	13	CDS	Yes	IV	2/2	No-PCR	**Yes**	No	**Yes**	HIX0026183	C11orf35
**20**	65	HIT000082518	3	11	CDS	Yes	I	1/9	**Spliced**	**Yes**	No	**Yes**	HIX0202311	PDIA2
**21**	65	HIT000252921	4	4	CDS	Yes	I	6/8	**Spliced**	**Yes**	UC	**Yes**	HIX0028549	TNFRSF18
**22**	65	HIT000058190	7	26	CDS	Yes	I	4/4	**Spliced**	**Yes**	**Yes**	**Yes**	HIX0039022	ADAM11

^a^ Serial number (SN). The 15 ultra-short introns that were confirmed are highlighted in bold font (see “Confirmation”); ^b^ Intron length within the ultra-short range (≤65 nt); ^c^ H-InvDB transcript (HIT) identifier; ^d^ Intron number (position) in the host gene (in the HITs); ^e^ Total number of introns (in the HITs); ^f^ Whether or not the intron is also found in the Ensembl transcript database (“Yes” or “No”); ^g^ Levels of sequence similarity of the encoded amino-acids (AA) sequence to known proteins or protein domains; ^h^ Intron frequency in the aligned HITs represented by the ratio of the number of HITs spliced at the ultra-short introns to the number of all aligned HITs across the ultra-short intron region; ^i^ The RT–PCR detection of the endogenous splicing or transcription. “**Spliced**”: splicing of endogenous ultra-short intron was observed. “Expressed”: splicing was not observed but transcription was observed; “No-Exp”: expression was not detected by RT–PCR but genomic PCR worked properly. “No-PCR”: PCR did not work, even with genomic DNA. “n/a”: RT–PCR could not performed because of the difficulty in designing a primer for the repetitive region; ^j^ Whether splicing of the ultra-short introns could be checked in mRNA-Seq data (“**Yes**”) or not (“No”); ^k^ Whether the transcripts were cloned and sequenced individually by researchers in the INSDC databases (“**Yes**”) or whether they just automatically sequenced in high-throughput studies (“No”). “RM” indicates that the original accession data were removed by the contributors, and “UC” indicates that the description was unclear. In the case of SN12, the original accession data were removed (CR600025 in INSDC), but the individually sequenced data were proposed by another source (D86358 in INSDC); ^l^ Confirmation of the ultra-short introns if at least one of three experimental studies (labeled with *) is positive (“**Yes**”), otherwise no evidence (“No”); ^m^ H-Invitational cluster (locus, HIX) identifier; ^n^ Approved gene symbols by HUGO Gene Nomenclature Committee (HGNC).

### 2.3. Confirmation of the Genuine Ultra-Short Introns by Database Analyses and Experiments

The authenticities of the 22 candidate ultra-short introns were further validated based on three criteria: (i) The presence of individually sequenced data in the International Nucleotide Sequence Database Collaboration (INSDC) databases (DDBJ/EMBL/GenBank); (ii) Verification with read sequences in the published mRNA-Seq data; and (iii) Our experimental verification by RT–PCR analysis of endogenous transcripts. Finally, we identified 15 ultra-short introns that fulfilled at least one of these three criteria (“SN” in Table 1, bold number).

(i) We inspected the original transcript data submitted to the INSDC database to judge whether the transcripts were cloned and sequenced individually at the specific interests of the investigators, or whether they were automatically sequenced in a high-throughput sequencing project. We found that eight introns were individually sequenced (“Individually sequenced” in Table 1).

**Figure 2:**
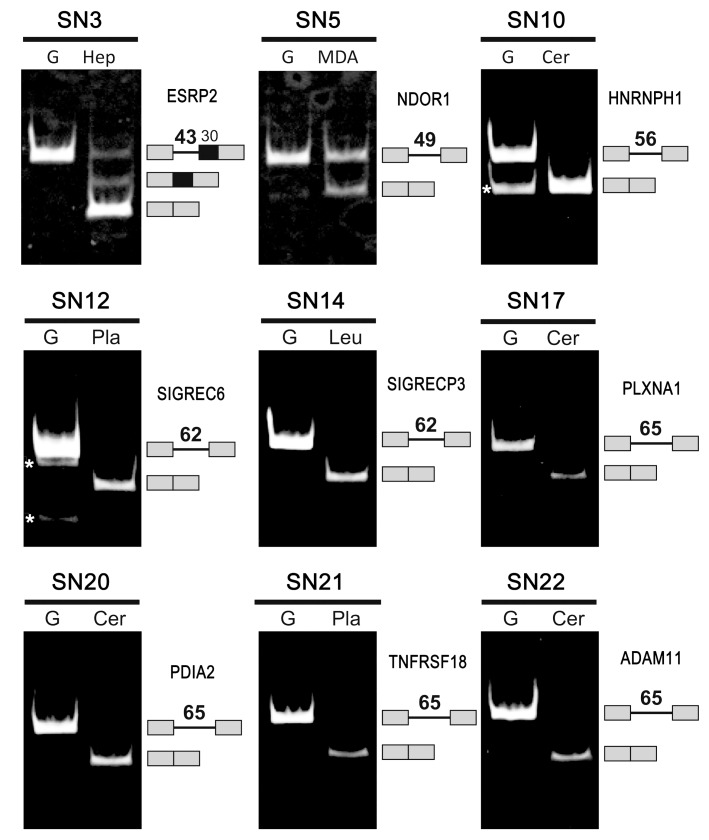
Splicing of 9 human ultra-short introns was detected in the indicated human cells (Hep, HepG2 cells; MDA, MDA-MB231 cells) and tissues (Cer, Cerebrum; Pla, Placenta; Leu, Leukocytes). See Table 1 for the gene names and serial numbers (SN). RT–PCR targeting the indicated endogenous gene transcripts was performed. G indicates the fragments that were amplified from genomic DNA. The amplified fragments corresponding to the pre-mRNAs and spliced mRNAs, separated by 5% PAGE, are indicated on the right with their schematic structures and the lengths of the introns (in nt). Asterisks (*) indicate nonspecific by-products that were not relevant to splicing. See Table S3 for more detailed information.

(ii) We confirmed whether the 22 ultra-short introns are spliced out by searching the publicly available RNA-Seq database of healthy human tissues—A total of 20 samples from 18 tissues; 16 tissues from BodyMap 2.0 project (see Section 3.3) and an additional four datasets of brain, pancreatic islet, stomach, and leukocyte samples (Supplementary Table S1). Our assembly of these RNA-Seq data demonstrated splicing at the junctions of the ultra-short introns in 12 ultra-short introns (“RNA-Seq data” in Table 1), some of which undergo tissue-specific splicing while others undergo ubiquitous splicing, such as the typical intron, SN10 (a 56-nt intron in HNRNPH1), which was spliced in all of the tissue samples (Table S1).

(iii) We performed RT–PCR experiments to examine whether the selected ultra-short introns are actually spliced out by endogenous splicing in human tissues. These experiments demonstrated that nine ultra-short introns were spliced out from the endogenous transcripts (Figure 2, Bold “**Spliced**” in Table 1). Splicing of the mini-gene transcripts containing three introns, SN3 (43 nt in ESRP2), SN5 (49 nt in NDOR1) and SN10 (56 nt in HNRNPH1), was previously confirmed in human cells and *in vitro* [8]. Of the other 13 transcripts, nine ultra-short introns were detected but they were not spliced (“Expressed” in Table 1), one ultra-short intron was not apparently expressed in the examined tissues (“No-Exp” in Table 1), and we could not obtain RT–PCR data for three ultra-short introns (“No-PCR” and “n/a” in Table 1). Although we did not perform RT–PCR in all tissues/cells for every transcript (22 introns), the results showed evidence of tissue-specific expression and splicing (Table S2).

### 2.4. Evidence to Support the Existence of Ultra-Short Introns

The authenticity of these selected 22 ultra-short introns was further investigated by screening the transcribed RNAs and the translated proteins of the host genes. The H-InvDB transcripts corresponding to the ultra-short introns are also listed in the Ensembl database, except for two transcripts, SN8 and SN9, which could not be identified in the Ensembl database (“Data in Ensembl” in Table 1). Eight introns could be identified with multiple transcript sequences spliced at the corresponding spliced junction in H-InvDB. Of these, four introns (SN14, SN15, SN19, SN22) and three introns (SN10, SN12, SN21) were spliced out in 100% and >75% of transcripts, respectively, whereas the other one (SN5) was spliced out in ~27% of transcripts, suggesting it is an alternatively spliced isoform (“Intron frequency” in Table 1).

To confirm the authenticity of these ultra-short introns, it is also important to determine whether the host genes of these introns encode authentic protein products. The H-InvDB classified the transcripts based on their similarities with known peptide sequences. The encoded proteins for 17 host genes harboring ultra-short introns were classified into the two highest categories that are either identical or similar to a known human protein (categories I and II in Table 1). These findings suggest that the correct splicing of these transcripts, or removal of the ultra-short introns, is essential to produce known functional proteins. Therefore, we examined the expected effects of the retention of these ultra-short introns caused by splicing defects (Table S3).

Of the selected 22 ultra-short introns, two introns (SN11 and SN16) were located within the untranslated region (UTR) while the other 20 ultra-short introns were located within the coding sequences (CDS; Table 1). Of these 20 introns, the lengths of three introns were multiples of three (54 nt for SN7 and SN8; 63 nt for SN15) with no risk of frame-shifting, but one intron (SN8) harbored a termination codon within the intron. Therefore, 17 ultra-short introns, if they are retained, could cause frame-shifts in the CDS (SN1–6, SN9, SN10, SN12–14, and SN17–22). Using these retained introns, we examined the position of the generating premature termination codon (PTC), which could be a potential target of the NMD, being located >50–55 nt upstream from the 3′-most intron or exon–exon junction (reviewed in [11,12]). Only three introns were excluded (the PTC downstream of the 3′-most exon–exon junction in SN2 and SN5; no PTC in SN21). Taken together, these results suggest that 15 introns are possible NMD targets (SN1, SN3, SN4, SN6, SN8–10, SN12–14, SN17–20, and SN22) if they retained in the mRNAs (Table S3). Our results further support the authenticity of these endogenous ultra-short introns because splicing of these introns is a prerequisite to produce functional mRNAs or proteins.

### 2.5. Unorthodox Sequence Features in the Ultra-Short Introns

We found that the G-content in conserved human introns gradually increases from the short range (85 nt) to the ultra-short range (≤65 nt; Supplementary Figure S1), which is consistent with the observation that the GC-content is higher in the shorter human introns [2,9,13,14]. Indeed, G was the most frequent base in more than half (12 in 22) of the ultra-short introns (underlined bold numbers in Table 2). All of the 12 G-rich introns contained repeated Gs, G-triplets or longer (SN1, SN3, SN5, SN7, SN12, SN14, and SN17–22). We previously identified a specific, 11-nt G-rich motif (CAGGGGCTGGG) in 43-nt (SN3; ESRP2) and 49-nt (SN5; NDOR1) introns that functions as an intronic splicing enhancer (ISE) [8]. We found highly homologous sequences with this ISE in another two ultra-short introns (SN12 and SN19; boxed sequences in Table 2). On the other hand, we also found apparent non-G-rich introns, occasionally with canonical branch-site sequences followed by the pyrimidine-tract (e.g., SN10, SN13, and SN16; underlined sequences in Table 2).

The essential core splicing signals (5′ splice site, 3′ splice site, and branch site) in the listed ultra-short introns were evaluated using the SROOGLE Web tool (see Section 3.5). Although we selected 22 ultra-short introns from among the candidate sequences with authentic GT–AG ends, none of the ultra-short introns had strong signals in all three splicing signals (Table 2). Surprisingly, several of the introns had scores for each site 0 or close to 0 (red numbers in Table 2; SN2, SN7–9, and SN15 for the 5′ splice sites; SN1, SN3, SN5, SN15, SN19, SN20, and SN22 for the branch sites; and SN1–5, SN8, SN11, SN15, and SN20 for the 3′ splice sites). Therefore, these introns can scarcely function as splicing signals under the known conventional splicing mechanism. Nevertheless, the RT–PCR assays demonstrated that some of these introns (SN3, SN5, SN20, and SN22) were indeed spliced out (Figure 2). The lack of these essential signal sequences, or the hallmark of authentic mammalian introns [15], is an unorthodox sequence feature in a major subset of these ultra-short introns.

**Table 2:** Sequence analyses and scoring of essential splicing signals of selected ultra-short introns.

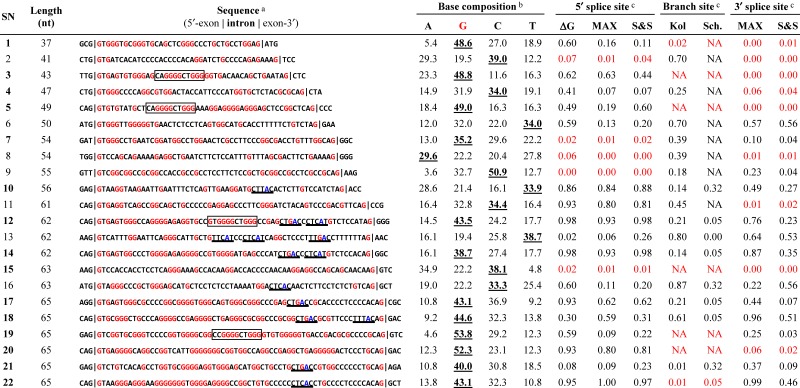

See Table 1 for the introns and their corresponding serial numbers (SN; bold numbers are the confirmed introns). ^a^ The consensus human branch site sequences, (C/T)TNA(C/T) [16], are underlined. The G nucleotide and the branched A nucleotide are highlighted in red and blue, respectively. Previously identified G-rich ISSs [8] are indicated with boxes; ^b^ The most frequent bases are indicated with underlined bold font; ^c^ The 5′ splice site, 3′ splice site, and branch site sequences were scored by SRROGLE [17]; ΔG (free energy of the base pairing with U1 snRNA), MAX (maximum entropy model) and S&S (Shapiro and Senapathy score), Kol (human-mouse comparative analysis) and Sch. (large-scale comparative analysis in eukaryotes). The detailed estimations of these algorithms have been reported elsewhere [18,19]. “NA” indicates that the value was not available due to lack of a target sequence. The inefficient splice sites and branch sites, with scores of <0.1 or “NA” for all the values in each pair/triplet, are highlighted in red.

Using the SROOGLE tool, we also searched for various exonic splicing enhancer (ESE) sequences in upstream and downstream of the ultra-short introns, which would potentially underpin nearby suboptimal, or weak, splice sites (reviewed in [20,21]). There was no significant difference in the contents and abundance of nearby ESEs between the ultra-short introns with efficient splice sites and those with inefficient splice sites (data can be provided upon request), suggesting that the known ESEs could not account for splicing of the ultra-short introns with defective splice sites.

### 2.6. Splicing Mechanism of the Ultra-Short Introns

The evident steric hindrance of the ultra-short introns together with the unorthodox sequence features suggests the presence of multiple splicing mechanisms that are distinct from the authentic spliceosome-dependent mechanism of action (reviewed in [4,5]). Here, we propose several clues to solve this intriguing question.

(i) Splicing of ultra-short intron *via* nonfunctional splice sites and branch site indicates that the splicing reaction occurs without either U1 or U2 snRNP. U1 snRNP-independent splicing was reported to occur under conditions of enriched SR proteins [22,23] or in particular pre-mRNAs [24,25]. This hypothesis is consistent with electron microscopic observation that *Drosophila* spliceosome with a shorter intron (62 *vs.* 147 nt as the control) have a smaller head domain containing the early “A” complex, suggesting that the complex with a shorter intron consists of fewer splicing factors [26].

(ii) The G-rich ISEs, instead of containing authentic splice site and branch-site sequences, could be critical for the recognition of ultra-short introns for splicing. We have demonstrated that a specific ISE, CAGGGGCTGGG, is essential to splice out two ultra-short introns (SN3 and SN5) [8], and we found another two introns (SN12 and SN19) that also include very similar sequences. Notably, this ISE contains two copies of G-triplets that often function as core sequences of ISEs to activate the upstream 5′ splice sites [27,28,29,30,31]. A subset, but not all, of the G-rich ultra-short introns must be spliced out with a specific *trans*-acting factor that recognizes a particular ISE. The 5′ splice site activation mechanism in which the G-triplet interacts with U1 snRNP *via* non-canonical base pairing with the U1 snRNA [32] might be involved in the splicing of G-rich ultra-short introns with an inefficient 5′ splice site. Consistent with this speculation, our preliminary *in vivo* splicing studies in HeLa cells showed partial splicing depression of G-rich 49-nt (SN5; NDOR1) and 43-nt (SN3; ESRP2) introns by disruption of U1 snRNA (unpublished data). The non-canonical U1 snRNA binding in these two introns (SN5 and SN3) remains to be analyzed.

(iii) A curious but important question is whether the G-rich ultra-short intron, which lacks an authentic branch site, is excised as a canonical lariat structure or not. We previously showed that a 56-nt intron (SN10; HNRNP H1) containing effective 5′ and 3′ splice sites and a branch site (Table 2) was excised as a lariat structure *in vitro* [8]. However, we could not detect the excised G-rich 49-nt (SN5; NDOR1) and 43-nt (SN3; ESRP2) lariat introns, which lack functional 3′ splice sites and branch sites (Table 2). The RNA structures of the excised ultra-short introns remain to be elucidated. It is not surprising to raise the issue of non-spliceosomal splicing, or even simple enzyme-catalyzed splicing.

## 3. Experimental Procedures

### 3.1. Extraction of Human Introns and Calculation of Intron Lengths

An integrated database of human genes and transcripts, H-InvDB Web server (version 6.0; http://hinv.jp/) [33], was used to obtain the coordinates of the intron–exon junctions. We only used the H-InvDB transcripts (HITs) based on the annotations on transcript sequences submitted to the INSDC databases (http://www.insdc.org/). Therefore, we used all of the human mRNA/cDNA sequences in the INSDC, except for computationally generated transcripts (pHITs and eHITs).

The sequences of the candidate introns were extracted from the human genome reference sequence (NCBI build 36; ftp://ftp.ncbi.nih.gov/genomes/H_sapiens/ARCHIVE/BUILD.36.3/Assembled_chromosomes/). Candidate intron sequences lacking a terminal GT and AG (the so-called GT–AG rule) were eliminated.

### 3.2. Selection of Ultra-Short Introns According to the Length and Conservation Status

To select unequivocal introns in the short range (*i.e.*, ≤85 nt), we performed three screening steps using 4528 tentative introns retrieved from H-InvDB. The first BLAST searches against the human transcriptome were queried by exonic sequences derived from genome coordinates of H-InvDB annotation. We could accordingly eliminate the H-InvDB transcripts that were erroneously mapped to genomic sequences with insertion/deletion generated by over-fitting to the GT–AG rule in the automated H-InvDB annotation process. This process yielded 4027 candidate introns, which were then screened by two kinds of BLAST searches: (i) NCBI Transcript Reference Sequences were queried for concatenation of both flanking exons, and (ii) Genomic Reference Sequences were queried for introns with both flanking exon sequences. These procedures were applied to each intron with the following thresholds: Length of obtained sequence >55; Bit score >99; *E*-value ≤1×10^−19^; Identity ≥96%.

If the ID of HIT, the intron number, and the species names were identical in both BLAST searches, we designated as conserved introns, which include 1253 introns in 35–87 nt and 23 introns in 37–65 nt. We removed one ultra-short intron sequence that was found to be a partial transcript sequence without an UTR, and obtained 22 introns as conserved ultra-short introns (Table 1).

### 3.3. Validation of the Ultra-Short Introns by Database Searches and Experiments

Given the fact that sequencing as an individual project is more reliable than sequencing as a part of comprehensive project using automated high-throughput screening methods, the 22 selected conserved introns (37–65 nt) were first checked against the source of the transcript sequences described in the INSDC database.

To gather evidence for the splicing of the 22 selected introns, we examined the following mRNA-Seq datasets. The BodyMap (2.0) project was searched via the Ensembl browser (http://www.ensembl. info/blog/2011/05/24/human-bodymap-2-0-data-from-illumina/). This dataset contains mRNA-Seq data from 16 human tissues, including adrenal, adipose, brain, breast, colon, heart, kidney, liver, lung, lymph, ovary, prostate, skeletal muscle, testes, thyroid, and white blood cells. We also investigated the sequence read archive databases (http://www.insdc.org/), which contain mRNA sequences derived from healthy human tissues: Brain (SRP002274), pancreatic islet (SRP010483), gastric tissue (SRP012016), and white blood cells (ERP000177). We generated index FASTA files for exonic fragments with defined lengths across each splice junction of the 22 ultra-short introns using ERANGE (getsplicefa.py program) [34], and then mapped the above mRNA-Seq data to the index FASTA file using Bowtie (version 0.12.8) [35].

We experimentally confirmed the splicing of these 22 introns using RT–PCR assays targeting endogenous gene transcripts. Total RNA extracted from human tissues (Takara Bio Inc., Otsu, Japan) was reverse transcribed with PrimeScript reverse transcriptase (Takara Bio) and oligo-dT primers. To detect the spliced products, the reverse-transcribed cDNAs were amplified by PCR with specific primers (Life Technologies, Tokyo, Japan; Table S4). The amplified DNA products were analyzed by 5% polyacrylamide gel electrophoresis (PAGE), as previously described [8].

### 3.4. Collection of Evidence to Support the Authenticity of the Ultra-Short Introns

We manually examined the 22 conserved ultra-short introns (≤65 nt) through H-InvDB (http://hinv.jp/; version 8.0), and collected annotated information for each intron and the hosted gene (first row in Table 1).

The frequency of the HITs spliced at the exact splice junctions corresponding to the ultra-short introns is represented as the ratio of the number of HITs spliced at the ultra-short introns to the number of all aligned HITs across the ultra-short intron region. We also checked for the presence of ultra-short introns in the Ensembl transcript database (http://www.ensembl.org/index.html). We finally identified the encoded proteins of the host genes using the curated results of H-Inv proteins, which were categorized based on the similarity between the predicted amino-acid sequence and the known sequences (http://hinv.jp/hinv/help/help_proteins.html).

### 3.5. Analyses of Sequence Features in the Ultra-Short Introns

The essential core splicing signals (5′ splice site, 3′ splice site, and branch site) of the 22 selected ultra-short introns were analyzed using SROOGLE Web server (http://sroogle.tau.ac.il) [17]. This integrated server detects and evaluates splicing signal motifs and generates scores based on several different built-in algorithms. The percentile scores relative to datasets of alternatively spliced exons (>3000) and constitutively spliced exons (>50,000) are provided [17].

## Supplementary Materials

Supplementary materials can be found at http://mdpi.com/1422-0067/16/05/10376/s1.
