# Supplementary material for: Identification and Validation of Evolutionarily Conserved Unusually Short Pre-mRNA Introns in the Human Genome
Source: Int J Mol Sci. 2015 May 7;16(5):10376–88. doi: 10.3390/ijms160510376 (PMC4463651; doi:10.3390/ijms160510376)
Supplement: Supplementary file 1 [file ijms-16-10376-s001.pdf]

Supplementary Information

Table S1. RNA-Seq data for human ultra-short introns.

**Studies**	**BodyMap 2.0 project**	**Individual mRNA-Seq studies [b]**			
**Study ID**	**ERP000546**	**SRP002274**	**SRP010483**	**SRP012016**	**ERP000177**
**Tissues**	**16 tissues [a]**	**Brain**	**Pancreatic islet [b]**	**Stomach [b]**	**White blood cell**
**No. reads**	**–**	**46824452**	**204819206**	**426101413**	**247858317**
**Introns (SN)**	**Splicing observed tissues**	**Number of reads across ultra-short intron**			
**1**	–	–	–	–	–
2	–	–	–	–	–
**3**	–	–	1	–	–
**4**	–	–	–	–	–
**5**	–	–	4	–	1
6	–	–	–	–	–
**7**	–	–	–	–	–
8	–	–	–	–	–
9	–	–	–	–	–
**10**	All 16 tissues	116	1826	12	1022
11	–	–	–	–	–
**12**	L	–	–	–	–
13	–	–	–	–	–
**14**	R, C, U, O, T, W	–	1	–	–
**15**	–	5	36	–	4
16	–	–	–	–	–
**17**	A, P, B, R, C, H, K, U, O, P, Y, T	26	28	–	2
**18**	W	–	2	–	3
**19**	A, R, K, M, P, Y, T	1	22	1	4
**20**	B, R	125	292	34	–
**21**	A, P, C, U, M, O, P, Y, W	5	1	–	9
**22**	B, H, O, P, T	43	–	–	1

See Table 1 for the introns and their serial numbers (SN; bold numbers are the confirmed introns). [a] Abbreviations used; A: adrenal; P: adipose; B: brain; R: breast; C: colon; H: heart; K: kidney; L: liver; U: lung; M: lymph; O: ovary; P: prostate; S: skeletal muscle; T: testes; Y: thyroid; W: white blood cells; [b] “Pancreatic islet” and “stomach” are not included in the BodyMap 2.0 project.

Table S2. Results of RT–PCR experiments using cells and tissues.

Introns (SN)	HIT ID number -intron no.	RT–PCR analysis [a]	Tissues or cells tested [b]
**1**	HIT000059291-1	Expressed	4 Cultured cells, Pancreas
2	HIT000276161-4	Expressed	Placenta
**3**	HIT000008845-6	**Spliced**	4 Cultured cells
**4**	HIT000325704-2	No-Exp	
**5**	HIT000009363-12	**Spliced**	4 Cultured cells
6	HIT000084762-8	Expressed	Cerebellum
**7**	HIT000325704-3	Expressed	Testis
8	HIT000333308-1	Expressed	Lung
9	HIT000278575-1	Expressed	Brain, Lung, Placenta
**10**	HIT000192494-7	**Spliced**	4 Cultured cells, 8 Tissues
11	HIT000302202-1	Expressed	Testis
**12**	HIT000279220-1	**Spliced**	Placenta
13	HIT000333305-1	Expressed	Brain, Cerebellum
**14**	HIT000495960-1	**Spliced**	Peripheral blood leukocyte
**15**	HIT000191419-3	n/a	
16	HIT000091849-1	Expressed	Testis, Placenta
**17**	HIT000324311-10	**Spliced**	Brain
**18**	HIT000058074-1	No-PCR	
**19**	HIT000052133-11	No-PCR	
**20**	HIT000082518-3	**Spliced**	Cerebellum
**21**	HIT000252921-4	**Spliced**	Lung, Cerebellum
**22**	HIT000058190-7	**Spliced**	Brain

See Table 1 for the introns with their serial numbers (SN; bold numbers are the confirmed introns). [a] RT–PCR detection of the endogenous spliced product or transcript. See Table 1 (footnote ‘i’) for the definitions; [b] Eight tissues were tested: Cerebrum (total brain), cerebellum, hippocampus, medulla oblongata, lung, peripheral blood leukocyte, placenta, and testis. Four cells were tested: Human mammary epithelial cells (HMEC); MCF7, MDA-MB231, and HepG2 cell lines.

Table S3. Potential NMD targets following retention of the ultra-short introns.

Introns SN [a]	Intron Length (nt) [a]	Intron position/ no. of introns [a]	Site of intron [a]	Effects of the retained intron			
				Frame-shift	Position of PTC generated		NMD target [b]
					From 3′ end of intron (nt downstream)	From 3′-most exon-exon junction (nt upstream) [b]	
**1**	37	1/3	CDS	Yes	51	515	**Yes**
2	41	4/4	CDS	Yes	34 (in 3′-most exon)	–34 (downstream)	No
**3**	43	6/14	CDS	Yes	(within intron)	1424	**Yes**
**4**	47	2/15	CDS	Yes	184	1805	**Yes**
**5**	49	12/13	CDS	Yes	426 (close to 3′-end)	–303 (downstream)	No
6	50	8/10	CDS	Yes	(within intron)	311	**Yes**
**7**	54	3/15	CDS	No	(no PTC)	(no PTC)	No
8	54	1/2	CDS	No	(within intron)	478	**Yes**
9	55	1/5	CDS	Yes	93	332	**Yes**
**10**	56	7/13	CDS	Yes	(within intron)	589	**Yes**
11	61	1/13	5'UTR	–	–	–	–
**12**	62	1/7	CDS	Yes	(within intron)	1120	**Yes**
13	62	1/2	CDS	Yes	(within intron)	92	**Yes**
**14**	62	1/6	CDS	Yes	(within intron)	935	**Yes**
**15**	63	3/4	CDS	No	(no PTC)	(no PTC)	No
16	63	1/2	5'UTR	–	–	–	–
**17**	65	10/28	CDS	Yes	(within intron)	2838	**Yes**
**18**	65	1/20	CDS	Yes	(within intron)	3453	**Yes**
**19**	65	11/13	CDS	Yes	224	173	**Yes**
**20**	65	3/11	CDS	Yes	97	1030	**Yes**
**21**	65	4/4	CDS	Yes	(no PTC)	(no PTC)	No
**22**	65	7/26	CDS	Yes	55	1629	**Yes**

[a] See Table 1 for the explanations with their serial numbers (SN; bold numbers are the confirmed introns); [b] If this number is >50–55 nt, it fulfills the criterion for NMD target (see text).

Table S4. Primer DNA sequences used in RT–PCR.

Target introns (SN)	Primer names (Forward & Reverse)	Primer sequences (5′ to 3′)
2	ENSA-F	CAAAAGTACTTTGACTCAGGAGACTACA
	ENSA-R	GACTAGGTGCCCATTTCATCCCTGCCTA
**3**	ESRP2-kozak-F	gccaccatggGTCAATTGTTTTCGAAGCCCGAGGTG
	ESRP2-R1	GCCACGGCAACCCACGAGCCCGTACC
**5**	NDOR1-kozak3-F	gccaccatggGCTGCCGCTGGCGGGACCAAGACTTC
	NDOR1-SalI-R	ggtcgacgAGGGCAGAGAACAGGGCTGGGGGC
6	SAMD14-F	GCCCAGCTCCTGGCTCATGCACAGGAGG
	SAMD14-R	TCTGAAGACTGAGACAGCGTGTGGTAGG
**7**	IFRD2-47nt-F	CCAAGTGCCCTGAGAAGCCAGTCCTGCC
	IFRD2-47nt-R	ATCCACGAGGCTAAAACTACTAATCCCA
	IFRD2-54nt-F	CTAGCCGCCGTCGCCTGCGCGCTCAGGC
	IFRD2-54nt-R	GGGGGGCGCGGGGTCAGGGACCCGGTGG
8	HSP90B2P-F	TCCAGTCTTCTCATCATCCAGCTGACAT
	HSP90B2P-R	CCTTCCTTGGCAACATTCTGGAACCTCT
9	AKIRIN2-F2	GCCACTCTGAAAAGGACTCTGGATTTCG
	AKIRIN2-R2	GAAGGGGGATGGCTCCATTCGGAGATAC
**10**	hnRNPH1-kozak-F	gccaccatggGCTATGGAGGCTATGATGATTACAATG
	hnRNPH1-SalI-R	ggtcgaCAGTAGCTCTGTAAGGTAATCCCCGC
11	MSTO1-F	ATTTTGCCGGTTTCGTGGGCGCGCACTG
	MSTO1-R1	GAGTCGCGGCGTGTAGGTCTCCTGGCCG
	MSTO1-R2	CTGGGGAAGCAGAGAGGACGAGGCAGGC
**12**	SIGLEC6-F	CCTCCACTCCCTTCCCCTTCTCTGCTCA
	SIGLEC6-R	GGCAATCTGCAGGGTACGAGGACGCACA
13	HSP90AB4P-F	CTGATGACATCACCCAGGAAGAGTGTGG
	HSP90AB4P-R	ACGGACAAAGCCGACATACTCTGGCATC
**14**	SIGLECP3-F	CACACAGGAAGCCCAGGAAGCCTCTGCC
	SIGLECP3-R	TTCCAGCCATACTCGAGGTAGAAGACCG
16	HIT000091849-F	GGAGAATAGAAATGCATTTTCTCTAAAC
	HIT000091849-R	CATTGGTGGTCACTCTGAGTTTTAGTTC
**17**	PLXNA1-F	TACTCCTACGAGGGGAACGATGTCAGCG
	PLXNA1-R	GCCACGCACCATCCGCACTCGAAGCGCG
**18**	RECQL4-F	ATGGAGCGGCTGCGGGACGTGCGGGAGC
	RECQL4-R	CGCGGGTCTCCTCCGGCGCCGCCTCCAC
**19**	C11orf35-F	AGAAGCCGCTGCGCGCGTCCTCGAGCCG
	C11orf35-R	GAGCGGGAAGCGGTCGATGGACAAGTCG
**20**	ARHGDIG-F	GCGCGAGCTGGCTGAGGAGTTTGGTGTG
	ARHGDIG-R	GCGCCTGGGCGGCCGCCTCGTCCTCCAG
**21**	TNFRSF18-F	ACTGCACATCTGGCAGCTGAGGAGTCAGTG
	TNFRSF18-R	CACCCACAGGTCTCCCAGCCGCCCCTTC
**22**	ADAM11-F	GGACCCCTTCCCCACCTCATTTACCGGA
	ADAM11-R	CTGCCTTTTCCTTCTCAGCCTCGGCCGG

See Table 1 for the introns and their corresponding serial numbers (SN; bold numbers are the confirmed introns). The inserted sequences (Kozak sequence and SalI site sequence) are indicated in lower-case letters.

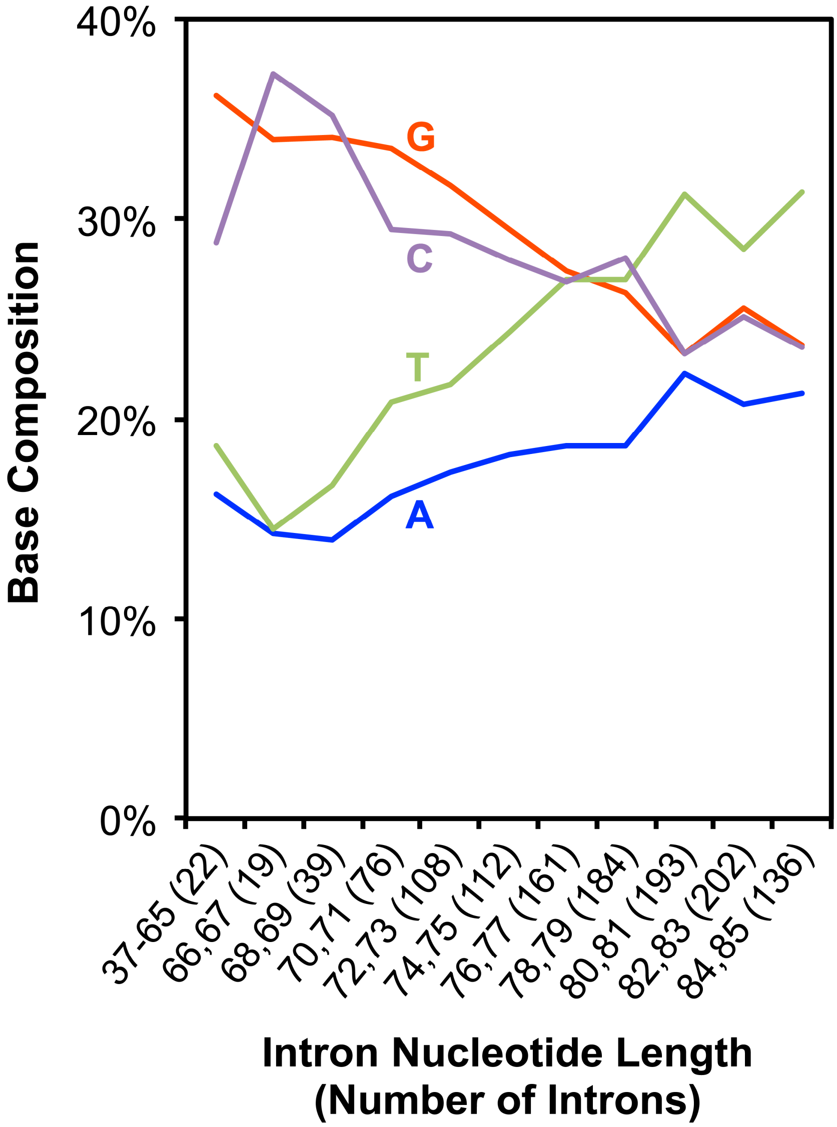

Figure S1. The G-base frequency shows a gradual increase in human short introns from 85 nt toward ≤65 nt. The average base compositions of the screened conserved human introns (total of 1252 introns; see section 3.2) were calculated and plotted in the ultra-short range (37–65 nt) and in the short range (66–85 nt) for each 2-nucleotide length. The number of short introns in each group is shown in parentheses.
